# Universal parent-focused child sexual abuse prevention: A quasi-experimental protocol

**DOI:** 10.1371/journal.pone.0314459

**Published:** 2025-01-16

**Authors:** Ella Abourjaily, Kate Guastaferro, Kassidy McElwee, Christian M. Connell

**Affiliations:** 1 Department of Social and Behavioral Sciences, School of Global Public Health, New York University, New York, NY, United States of America; 2 Child Maltreatment Solutions Network, Social Science Research Institute, The Pennsylvania State University, University Park, PA, United States of America; 3 Human Development and Family Studies, College of Health and Human Development, The Pennsylvania State University, University Park, PA, United States of America; Universitair Kinderziekenhuis Koningin Fabiola: Hopital Universitaire des Enfants Reine Fabiola, BELGIUM

## Abstract

**Background:**

Child sexual abuse (CSA) is a significant public health concern, and there is a lack of universal, evidence-based primary prevention interventions that extend beyond a focus solely on children. Parents remain a consistently underutilized target for primary prevention efforts aimed at mitigating CSA despite their unique relationship and close proximity to their children. CSA risk is not confined to any specific demographic, and its effects on affected children are well-documented, significantly impacting numerous dimensions of their wellbeing. Thus, there is a clear and urgent need to address this gap in prevention strategies.

**Methods:**

This study will use a quasi-experimental design (target *N* = 412) to examine potential gains in CSA-related awareness and intentions to use protective behaviors among parents who participate in a universal parent-focused CSA prevention workshop, *Smarter Parents*. *Safer Kids*., compared to those who do not. Participants in both the control (*n* = 206) and experimental group (*n* = 206) will complete 3 survey assessments: Survey 0 (baseline), Survey 1 (1-month), and Survey 2 (3-month follow-up). The experimental group will participate in a *Smarter Parents*. *Safer Kids*. workshop between the Surveys 0 and 1. We will use data collected from the baseline to measure potential mediators of CSA-related awareness and intention to use protective and preventive behaviors. In adjacent efforts to enhance the curriculum’s reach with future dissemination and implementation, we will also explore the impact of recruitment materials and strategies on parental engagement.

**Conclusion:**

Results of this study will advance efforts to implement parent-focused CSA prevention with a universal audience.

## 1. Introduction

### 1.1. Background and rationale

Child sexual abuse (CSA) is an urgent public health concern estimated to impact over 60,000 children each year in the United States [1, 2]. It is widely accepted, however, that this is a gross underestimate of the true prevalence of CSA due to barriers in disclosure by children (e.g., the vocabulary required to disclose; an understanding of the acts that took place; access to supportive resources and trusted personnel; and fear of retribution, removal from the home, not being believed, social stigma and/or shame) [3, 4]. Evidence of this underrepresentation is displayed in the most recent report by the National Children’s Alliance indicating that while not all culminated in disclosure, 236,601 cases of CSA were opened and investigated by U.S. child advocacy centers in 2023 [5]. Consequences of CSA include lifelong adverse physical, psychological, social and behavioral health outcomes as well as an increased risk of subsequent revictimization [6], resulting in an estimated lifetime economic burden in excess of $9.3 billion USD for 1-year of substantiated victims [7]. Given the considerable magnitude of CSA and recognizing that CSA is a preventable issue [8], an increase in primary universal prevention efforts is urgently needed.

For years, CSA interventions adopted secondary and tertiary prevention approaches [9]–with a focus on streamlining efforts towards mitigating adverse outcomes of CSA after it has occurred and preventing revictimization. While important, this leaves a gap in preventing CSA from occurring in the first place–known as primary prevention [9–11]. The majority of extant evidence-based CSA primary prevention programs are largely child-focused and delivered in schools [12–14]. However, the field agrees that children should not bear the responsibility of protecting themselves from victimization. Parents (or primary caregivers) have long been neglected in primary prevention efforts for CSA [15], an indication of the unique etiology in comparison to other forms of maltreatment and the knowledge that parents are not often the primary perpetrators (36%) [16]. Parents are in an optimal position to communicate with and protect their children from harm, given both their unique physical proximity (i.e., monitoring) and the inherent nature of a parent-child relationship (i.e., communication, scaffolding of social norms) [10]. In a systematic review of 24 parent-focused CSA prevention programs, few studies (n = 7) used a randomized controlled trial design indicating a need in the evidence-base of these programs [17]. Improvement was most commonly observed related to parental behavioral intentions and response efficacy indicating a need to focus more on actual use of protective behaviors. Importantly, the review included no universal parent-focused CSA prevention efforts.

### 1.2. Parent-focused CSA prevention

Our team previously developed a parent-focused CSA prevention curriculum, *Smart Parents–Safe and Healthy Kids* [18, 19], which was designed to be an added session to parent-support programs. In this way, the curriculum adopted a selective approach to prevention [20] as children of parents enrolled in parent-support programs (e.g., home visitation programs) are seen to be at a heightened risk for CSA [21–23]. However, CSA affects children across all sexes, ages, races, ethnicities, and socioeconomic stratum. Therefore, a need remains for an evidence-based universal parent-focused CSA prevention curriculum.

Adapted from the selective intervention [19], we created *Smarter Parents*. *Safer Kids*. (hereafter *Smarter Parents*) to be a universal parent-focused CSA prevention curriculum which is delivered in a two-hour, single session group-based workshop for parents of children under 13-years old [24]. Beyond the age of 13, there enters a different level of complexity when adding in other levels of sexual behavior (e.g., dating) that arise after the onset of puberty. Thus, the curriculum maintains a focus on children under 13 –addressing the age group most vulnerable to CSA [25, 26]. As in the selective intervention, the universal *Smarter Parents* curriculum is behaviorally based and provides parents with developmentally appropriate information on three sections related to reducing risk for CSA: (a) healthy child sexual development; (b) parent-child communication about sex, sexual development, and sexual abuse; and (c) child safety (e.g., vetting babysitters, monitoring activities inside/outside the home and online). In the workshop, facilitators use structured discussions and interactive role-playing scenarios to practice the skills and strategies taught (i.e., “Your 3-year-old son saw your 4-year-old daughter naked after taking a bath. He says, ‘Where is sister’s wee-wee?’ How would you respond?”).

The *Smarter Parents* curriculum was pilot tested using a mixed methods approach to examine acceptability and feasibility [24]. Parent participants (*N* = 31) attended a workshop and completed pre- and postworkshop assessments measuring CSA knowledge and intention to use protective behaviors. Participants also engaged in a brief structured interview with the research team after the workshop. Mean post-workshop assessment scores increased meaningfully from pre-workshop assessment, and qualitative data indicated high acceptability. The pilot was limited by its small and homogeneous sample along with its lack of a control group [27]; however, results indicate promise for evaluating the effectiveness of the *Smarter Parents* curriculum in future research.

### 1.3. Current study

Next in the line of research to establish an evidence base for *Smarter Parents*, the current study will capitalize on the active implementation of *Smarter Parents* by nine community-based partners across the Commonwealth of Pennsylvania–referred to as Project Safe and Smart. This study will therefore use a quasi-experimental design (target *N* = 412) to examine potential gains in CSA-related awareness (i.e., knowledge of and attitudes toward appropriate CSA prevention strategies) and intentions to use protective behaviors among parents who participate in *Smarter Parents* compared to those who do not (Aim 1). Then, we will consider potential mediators of CSA-related awareness and protective behaviors (e.g., demographic characteristics, initial parenting strategies, use of protective and preventive behaviors; Aim 2). Lastly, we will also explore the impact of recruitment materials and strategies on parental engagement (i.e., workshop registration and attendance; Aim 3). Results of this study will contribute to the growing evidence base of *Smarter Parents* and will advance efforts to implement universal CSA prevention with parents and primary caregivers.

## 2. Methods

### 2.1. Workshop implementation

Implementation of the workshop by the nine community-based organizations is supported by grants awarded by a Pennsylvania state-government agency. Selected via competitive application process the sites represent victims service agencies, child advocacy centers, and youth serving organizations. Facilitators (*n* = 2 per site) were trained to deliver the *Smarter Parents* workshop in August of 2023. Once certified, facilitators could train other facilitators at their site to deliver the workshop; certification for all facilitators was provided by the research team. Implementation began in earnest across all sites in early 2024.

As designed, the group-based *Smarter Parents* workshop may be delivered in-person (i.e., at the site, a library, or other community location) or online (via interactive videoconferencing software such as Zoom). To achieve model fidelity, it is required each workshop follow a 1:10 ratio wherein there is 1 facilitator for every 10 participants (max. 20 participants). Parent participants will receive a physical copy of the *Smarter Parents* Parent Handbook at in-person workshops, or an electronic copy (i.e., PDF sent via email) for online workshops. Facilitators will submit an implementation form for each scheduled workshop regardless of the number of registrants or participants. This form documents the date and time of the workshop, number of registrants, number of participants, aspects of fidelity, rating of participant engagement, and documentation of any unforeseen circumstances (e.g., disclosure, interruption, etc.). Facilitators are encouraged to attend monthly learning collaborative style implementation meetings held to highlight successes and address any challenges or concerns that have risen for facilitators during implementation processes. As this is an active implementation, sites are responsible for marketing and advertising. Per their grants, sites are able to provide meals, transportation, childcare, or incentives (e.g., gift cards, gas cards) for workshop participants. All marketing and promotional materials will be sent to the research team on a quarterly basis for examination in Aim 3 of the current study.

### 2.2. Participants

Eligibility criteria will require participants to be over the age of 18, English speaking (i.e., native or fluent), a Pennsylvania resident (verified by zip code), and a parent of at least one child under 13 years of age. Participants must also have internet access to complete the experimental procedures. For the purpose of this research, the term “parent” is loosely defined to include any primary caregiver of a child under 13-years old including, but not limited to, birth or biological parents, stepparents, foster parents, or grandparents. Screening for eligibility will be conducted through designated REDCap fields that ask participants to self-confirm that they meet criteria. Additional screening for bots (i.e., non-human respondents) will be conducted by the research team using “human” questions in Survey 0 (e.g., “Are you a human?”, “What color is a lemon?”) as well as a logic question (e.g., “Rachel thanked Matilda for all the help she received. Who received help?”). We use a ReCAPTCHA tool as recommended in the literature [28] and learning from prior our experience [24], our team has identified common patterns of bot entries (e.g., multiple sequential registrations within seconds of each other, participants with two first names, zip codes outside of the eligible area) and will be manually flagged by the research team for review.

### 2.3. Experimental procedures

All procedures were approved by the Institutional Review Boards at New York University and the Pennsylvania State University. A waiver of written consent was obtained wherein if participants decided to move forward in the survey after viewing the consent form, they were providing their implied consent. Study recruitment launched on May 1, 2024, and the targeted date for finalizing recruitment is December 31, 2024. As randomization at the site level is not feasible, parents who participate in the *Smarter Parents* workshop with one of our 9 partner sites will comprise the experimental group (target *n* = 206). Control participants (i.e., those who do not receive the *Smarter Parents* workshop; target *n* = 206) will be recruited from generalized recruitment ads distributed by community partners in Pennsylvania (not engaged in Project Safe and Smart) at similar agencies to those providing the workshop and recruiting the experimental group.

**Fig 1:**
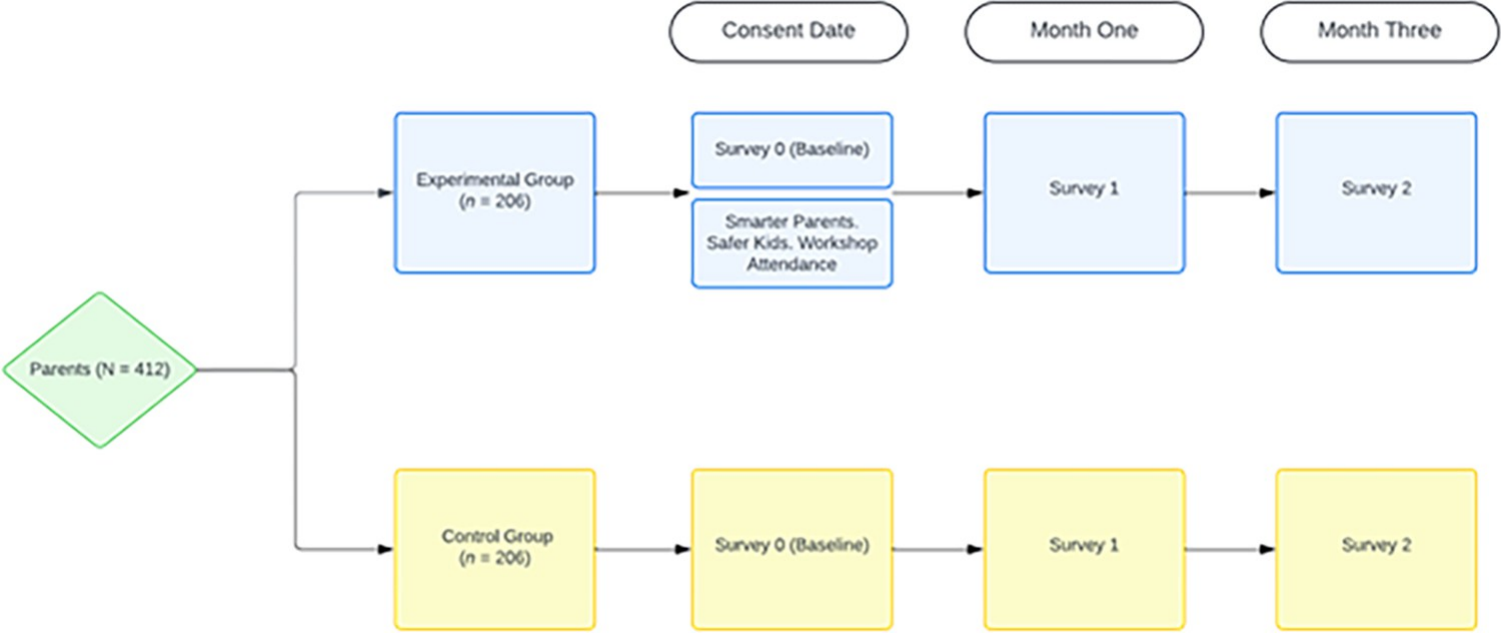
Study design.

Participants, whether experimental or control, will complete three 15-minute surveys administered on REDCap (Research Electronic Data Capture) [29]: (a) Survey 0 (i.e., baseline), upon registration for the research; (b) Survey 1, completed 1-month after Survey 0 and workshop attendance, where applicable; and (c) Survey 2, 3-month follow-up (Fig 1). Participants will scan a QR code located on the recruitment materials to bring them to the screening form which then automatically proceeds to the consent form and will be provided a copy for download. Advancing to the first survey indicates their implied consent. Each subsequent survey will be sent at the appropriate timepoint via unique REDCap link to the email address the participant provides in Survey 0. Email reminders to complete each survey will be sent every two days up to a total of five times per participant.

### 2.4. Measures

At Survey 0, participants will be asked to provide basic demographic characteristics (i.e., age, number of children, level of educational attainment). The primary outcome of interest, parents’ CSA-related awareness and parent behaviors will be assessed using the Assessment of SmartParents’ Knowledge (ASK) [18]. The 15-item ASK, rated on a 5-point Likert scale (i.e., Strongly Disagree to Strongly Agree), provides an indicator of parents’ CSA-related awareness (e.g., “Most sexual abuse victims are abused by someone they know”) and intention to use protective behaviors (e.g., “I have talked to my child about how to protect themselves from being sexually abused”).

To explore CSA-related preventive behaviors used by the parent, at Survey 0 all parents will be asked to complete the 10-item preventive behaviors questionnaire (PBQ) [30]. On the PBQ, parents to report how frequently in the past month (i.e., never [0], sometimes [1–5 times a month], or a lot [6–10 times a month]) they performed a named preventive behavior (e.g., “Educated [their] child about the difference between safe and unsafe touches” or “Modeled or talked about privacy in the home.”). The PBQ, thus, measures actual use of protective behaviors.

To examine general parenting modalities, all parents will be asked to complete the 16-item Knowledge of Parenting Strategies Scale (KOPSS) [31] at Surveys 0 and 1. The KOPSS asks parents a set of multiple-choice questions about parenting practices (e.g., “It is 5 minutes before dinner time and a child is throwing a tantrum for a cookie. What should mum do?”) and awards a parent one point per correct answer. Higher scores indicate a greater degree of use of desired parenting qualities.

There is a paucity of standardized measures to demonstrate actual use of protective behaviors. We will leverage this study to pilot a questionnaire format for the Parents Use of Behavioral Strategies Interview (PUBSI), a project generated assessment tool, at Survey 2. The 23-item assessment will evaluate parent use of protective behaviors using mixed response types including: yes or no questions; a 5-point Likert scale assessing confidence; and short answer textboxes. Examples of questions include “Have you made changes to your home environment to protect your child from sexual abuse?”, “How confident are you that your child knows who to go to (or talk to) if they feel unsafe?”, and “What names does your family use to refer to private parts?”. Change in the prevalence of CSA is the ultimate outcome of interest; however, the stigma of and mean time to disclosure [32–34] reduce the feasibility of using administrative data for outcomes in any CSA intervention research. In addition, verifying self-report accuracy is beyond the scope of this study. The outcome of interest in the current study is the increased use of CSA-related knowledge and self-reported use of protective behaviors.

Participants in the experimental group will also be asked to complete a satisfaction form on Survey 1 (delivered 1-month post-workshop, Fig 1). This form asks the parents about their perceptions on preventing CSA before and after the workshop as well as the acceptability of the program and how likely they are to recommend the *Smarter Parents* workshop to other parents. To understand the ways in which parents interacted with the workshop, parents will be asked to report the device used to join the workshop (i.e., desktop computer, laptop, tablet, etc.). Parents will also be asked to provide suggestions on how to make the workshop more accessible to caregivers and how to promote the workshop in their community (e.g., fliers in school, school-based apps, counselor recommendations, etc.).

### 2.5. Incentive structure

Participants in both groups will be eligible to receive up to $100 in Amazon e-gift cards distributed via participant-provided email. Participants in the control group will receive a $20 Amazon e-gift card after completion of Survey 0. Those in the experimental group will receive this $20 Amazon e-gift card after completion of Survey 0 and verified attendance in a *Smarter Parents* workshop provided by sites. After the completion of Survey 1, all participants will receive a $30 Amazon e-gift card and a $50 Amazon e-gift card after completion of Survey 2. This incremental incentive structure is a common approach implemented by our team to promote participant retention throughout the course of the study.

### 2.6. Statistical plan

#### 2.6.1. Sample size and power

Without a strong literature base to refer to for universal parent-focused CSA prevention programs, we used prior research of the selective parent-focused program [19] and a meta-analysis of parent-focused child maltreatment prevention interventions [35] to inform our effect size estimation. For the current quasi experimental study, we assumed a conservative pre-post correlation of .7, specified a small effect size of a Cohen’s *d* of .2, and set the measure of significance, α, to .10 (i.e., adopted decision-priority perspective) [36]. Using a power calculator [37], the results of our power calculation indicated a need for 317 total participants (158 per condition). However, accounting for an estimated 30% attrition (i.e., loss to follow-up), we will aim to recruit a total of *N* = 412 participants, *n =* 206 per condition.

#### 2.6.2. Analytic plan

Quantitative analyses of the resulting pre- and post-workshop data will examine whether levels of CSA-related awareness and intention to use protective behaviors are greater for parents who participated in a Smarter Parents workshop in comparison to those who did not. As we will have longitudinal quasi-experimental data, we will use a difference-in-difference (DID) approach to analysis [38]. DID is appropriate when (a) randomization at the individual level is not possible and (b) the goal is to examine the effect of a specific intervention by comparing changes overtime among those who received the intervention (experimental) and those who did not (control). Using the DID regression framework, meditation analyses will examine potential differential effects by parent characteristics (e.g., demographics, initial parenting strategies [KOPSS] and actual use of protective and preventive behaviors [PBQ]).

To understand the impact of recruitment materials on parental engagement, we will first qualitatively code the strategies used on recruitment materials provided by the sites (e.g., theoretical framing, image, testimonial, incentive, etc.). We will quantify which strategies were used most consistently across the experimental site. We will also map types of ads used by the sites to registration/attendance numbers reported on the implementation forms. Participant responses on the satisfaction form (e.g., “Do you have any suggestions on how to promote Smarter Parents in your community?”) will be coded thematically to inform sites recruitment efforts in the future. All data will be made available in the lead authors’ university data repository once the study is complete.

## 3. Conclusion

Though incidence rates of CSA in the U.S. have declined meaningfully since the 1990s [39], the most recent estimates suggest a prevalence rate of nearly 22% [40]. CSA remains a public health concern of considerable magnitude. Declining rates may be attributable to myriad CSA prevention efforts [41]; it is unlikely that a singular strategy focused on only one segment of the population will affect rates of CSA [42]. It is our belief that there is real potential to affect rates when parents and children alike have the tools to talk to each other. It is imperative that parents be included in primary prevention efforts [15]. The current study, while it does not use a randomized controlled trial design, advances the science of parent-focused CSA prevention by measuring actual use of protective behaviors and grows the evidence-base of universal CSA prevention efforts.

While this work was initially adapted from a selective prevention curriculum [19], universal prevention for parents regardless of risk is a crucial next step towards protecting all children from CSA. The perspective of the *Smarter Parents* curriculum, and this study, is that equipping all parents–regardless of risk level–with the knowledge and skills to protect their children from victimization is a crucial part of the prevention spectrum. It is understood that the nature of universal CSA prevention may introduce selection bias (i.e., wherein those at most risk may not opt to engage in such a program), however, this is inevitable with most any universal program and can be seen in selective prevention interventions as well [43, 44]. The benefits of universal prevention far outweigh its challenges [43]. Results of this quasi-experimental trial will contribute to the evidence base of *Smarter Parents*. *Safer Kids*. and take us one step closer to bringing parent-focused CSA prevention to a universal audience.
